# Physical activity participation in people with severe mental illness: The real challenge

**DOI:** 10.1002/gps3.70058

**Published:** 2026-09-15

**Authors:** Mikel L. Sáez de Asteasu, Simon Rosenbaum, Mikel Izquierdo

**Affiliations:** ^1^ Navarrabiomed Universidad Pública de Navarra (UPNA) Pamplona Spain; ^2^ CIBER of Frailty and Healthy Aging (CIBERFES) Instituto de Salud Carlos III Madrid Spain; ^3^ Nutrition, Exercise and Social Equity Research Group, Discipline of Psychiatry and Mental Health School of Clinical Medicine University of New South Wales Sydney New South Wales Australia

## CONFLICT OF INTEREST STATEMENT

The authors declare no conflicts of interest.

The third report from *The Lancet Psychiatry* Physical Health Commission is refreshingly blunt about the problem: people living with severe mental illness (SMI) face premature mortality, essentially from preventable physical diseases linked to modifiable risk factors such as tobacco use, low physical activity, poor diet quality and disrupted sleep.^1^ What has changed since the 2019 Commission is not the strength of the evidence on benefits (that argument is settled), but the insistence that we stop treating implementation as an afterthought. The uncomfortable truth is that lifestyle care in SMI has often been framed as ‘nice to have’, whereas pharmacotherapy and crisis management remain ‘must‐haves’. That hierarchy is clinically indefensible and operationally outdated. The central question is no longer whether physical activity should be part of SMI care, but how health systems make it routine, funded, staffed, measured and safe.^2^

Physical activity is the clearest example of the implementation gap regarding lifestyle interventions. The World Health Organization's population guideline of 150 min per week of moderate‐to‐vigorous physical activity (MVPA) is rarely reached in SMI; pooled estimates suggest that average MVPA is a fraction of that observed in the general population.^3^ This is not a failure of information—most patients have heard that exercise is ‘good for you’. It is a failure of service design. When physical inactivity is treated as an individual behavioural deficit rather than a predictable consequence of illness, medication effects, trauma exposure, poverty, stigma and fragmented care, we build programmes that look good on paper and quietly fail in real life. The cost of that failure is not abstract: cardiovascular disease remains the dominant driver of premature mortality in mental disorders,^4^ and inactivity compounds the metabolic risk already amplified by antipsychotic treatment.

If we want a fresher perspective, we should stop describing physical activity as a ‘behaviour to change’ and start treating it as a clinical capability that services must deliver, in the same way they deliver medication review, smoking cessation or diabetes prevention.^5^ Physical activity is an ‘adoption behaviour’ because it involves uptake and sustained practice, often without immediate reward, and it competes with powerful illness‐related barriers. Negative symptoms, low mood, anxiety, perceived stress, limited social support, physical comorbidity and lack of prior positive experiences all predict poor uptake.

Weight gain and sedation associated with psychotropic medication can make movement feel harder and less rewarding, particularly in the early weeks of treatment when motivation and self‐efficacy are fragile. These are not reasons to lower ambition; they are merely design constraints. A programme that does not explicitly account for these constraints will default to selecting those already fit and excluding those who most need it.

So, what does implementation look like when we take those constraints seriously? It begins with a simple shift: moving physical activity from an ‘optional adjunct’ to a ‘default component’ of care, with a minimum service standard. In practical terms, that means every patient should have (1) a brief physical activity assessment embedded into routine contact, (2) an agreed pathway to access supervised support when indicated and (3) follow‐up that treats continuation as a clinical outcome rather than a personal choice that sits outside the service's remit.

A workable pathway need not be complicated, but it must be explicit. At entry points—admission, intake, medication initiation, annual review—services can record a ‘physical activity vital sign’ alongside cardiometabolic risk, smoking, sleep and diet.^6^ The purpose is not surveillance; it is triage. A small set of triggers can guide supervision intensity: cardiometabolic risk burden, current functional limitation, fall risk, medication profile (e.g., sedation, orthostatic symptoms), exercise‐related adverse events (e.g., musculoskeletal injury, cardiovascular events in high‐risk patients) and patient preference and confidence. This immediately solves a common failure mode: offering the same intervention to everyone, then blaming ‘motivation’ when it does not stick. Some people can start with supported self‐management; others need a structured, supervised on‐ramp to make early experiences safe and rewarding.

Workforce is the next bottleneck. Many mental health teams already carry impossible workloads; adding ‘exercise counselling’ as another task without staffing changes guarantees superficial delivery. The Commission's direction points to a more realistic model: embed exercise professionals—clinical exercise physiologists, physiotherapists or appropriately trained practitioners—within mental health services, with clear interfaces to psychiatry, nursing, occupational therapy and pharmacy. The feasibility of this innovative framework will depend on funding and workforce availability. Implementation may be enabled through reimbursement pathways within existing mental health care settings and the incorporation of scalable workforce models. Services without funding to hire exercise professionals could initially use a train‐the‐trainer model, training occupational therapy or nursing staff in exercise prescription. Digital platforms could also offer an alternative means of delivering exercise interventions. Multidomain interventions, including exercise interventions, may be cost‐effective in patients with SMI, but further research is needed to compare the cost‐effectiveness of different exercise programmes (e.g., group‐based programmes versus individual sessions) and for different patient subgroups, such as patients with diverse psychiatric diagnoses, different genders and comorbidity status.^7^ This is not about importing a fitness model into psychiatry. It is about building a shared clinical language for dosing, progression, adverse effects and risk management. Exercise professionals working in SMI services need additional training in trauma‐informed care, de‐escalation, psychosis‐informed communication and medication‐related physiological effects.^8^ Conversely, prescribers need sufficient exercise pharmacology literacy to anticipate interactions and avoid preventable harms.

Those interactions are not hypothetical. Antipsychotics can increase appetite and weight, worsen insulin resistance, reduce subjective energy and alter thermoregulation; antidepressants and mood stabilisers can influence heart rate responses, hydration status, and perceived exertion; and polypharmacy can amplify orthostatic symptoms and fatigue.^9^ A patient starting clozapine or olanzapine may need a different entry dose and monitoring plan than a patient on a metabolically neutral regimen—not because exercise ‘doesn't work’, but because the early experience must be engineered to feel feasible. Timing matters, too: if sedation peaks after dosing, scheduling sessions when alertness is higher can be the difference between a reinforcing experience and a humiliating failure. In other words, implementation requires that exercise not be simply ‘recommended’ but be clinically co‐managed with pharmacotherapy.^10^

The content of programmes also needs reframing. Too often, services interpret physical activity as either a generic walking leaflet or an unrealistic gym‐based prescription. Long‐term adherence is more likely when activities satisfy psychological needs—autonomy, competence, relatedness—and when the patient experiences early mastery rather than early punishment. That is why autonomy‐supportive approaches grounded in motivational theory are not academic luxuries but core implementation tools.^1^ Self‐efficacy, action planning, coping strategies and autonomous motivation correlate positively with activity in mental disorders, whereas fear of negative experiences, low knowledge of ‘how to start’ and perceived barriers predict dropout. The practical implication is straightforward: programmes should be built around a ‘menu’ of options that allow preference‐based matching—walking groups, dance, resistance training, balance work, interval‐style microbouts, home‐based routines—paired with coaching that focuses on confidence and problem‐solving, not guilt.^11^ In line with the knowledge‐to‐action framework, well‐established implementation frameworks such as the Consolidated Framework for Implementation Research; the Reach, Effectiveness, Adoption, Implementation, Maintenance framework; and the Exploration, Preparation, Implementation, Sustainment model should be considered to create a robust approach for improving the adoption of an active lifestyle for mental illness prevention.^12^, ^13^, ^14^ Furthermore, the implementation of these programmes in low‐ and middle‐income countries is especially necessary to ensure a minimum viable service in under‐resourced community settings.^1^ In SMI, the smallest unit of success is not 150 min per week; it is repeating a manageable dose often enough that identity and routine start to shift.

This is also where services can stop unintentionally widening inequities. A pathway that assumes access to transport, disposable income, digital access, safe neighbourhoods and flexible schedules will primarily benefit people who are already advantaged. Equity must be engineered: funded transport vouchers, nondigital enrolment routes, culturally responsive sessions, women‐only or gender‐safe options where needed and supported onboarding for those with cognitive impairment or severe social anxiety. Group formats can be powerful for relatedness, but they are not universally safe; offering both individual and group options is not ‘extra’; it is basic accessibility. Heterogeneity in age, gender, symptom profile, trauma history and readiness to change is not noise—it is the clinical reality that implementation must accommodate.^1^

Community delivery is where many programmes collapse, because the handover from inpatient or outpatient care to real‐world environments is treated as the patient's responsibility. Yet community is precisely where sustained activity is won or lost. Health systems do not need to control urban design to act; they can commission partnerships. For example, community mental healthcare and peer support for discharge from inpatient psychiatric care, offered to people with previous admissions, can also reduce average costs of mental health care use without being detrimental to individuals.^15^ Social prescribing models, agreements with community centres, referral pathways to adapted sports clubs and co‐designed programmes with lived‐experience leadership can extend reach without having to recreate everything within hospitals. Initiatives such as Addi Moves in Sydney—a free, culturally responsive, trauma‐informed service supporting individuals affected by trauma, including those with lived experience of SMI—illustrate an important principle: trust is not an ingredient you sprinkle on top; it is the delivery mechanism.^16^ A total of 1306 one‐on‐one and 783 group sessions were provided through Addi Moves, with an average of 96% of participants opting to return.^16^ For many patients with SMI, ‘going to a gym’ is not just inconvenient—it can feel unsafe, exposing and stigmatising. Creating psychologically safe spaces is part of the intervention, not a backdrop.

None of this will scale without measurement and accountability. If exercise remains invisible in routine data, it will remain unfunded and fragile. Services should track a small set of implementation outcomes alongside clinical outcomes: referral rates, uptake, completion, adverse events and maintenance at follow‐up—stratified by key equity variables. Patient‐reported experience measures should include perceived autonomy support, confidence and safety. Importantly, missing data should be treated as a signal: if certain groups are consistently ‘lost to follow‐up’, this is more likely to reflect exclusion than randomness. These metrics are not about blaming clinicians but how systems learn what works, what fails and for whom.

This brings us to the ethical question: is it acceptable not to provide a structured pathway to promote exercise engagement among people with SMI?^17^ If we know that preventable physical disease is a leading cause of early death, and if physical activity is a core, evidence‐based countermeasure, then failing to provide a pathway to deliver it is not neutral but a structural omission. Aligning physical activity with rights‐based mental health policy is not rhetorical. It means treating physical health protection as a matter of dignity, rather than a discretionary add‐on for those who can manage it.

The Commission's call to pivot towards implementation research is timely because the research agenda now needs to change. We do not need another efficacy trial showing that exercise improves symptoms or cardiometabolic markers. We need pragmatic trials and natural experiments that compare delivery models: embedded exercise professionals versus referral‐only models; supervised ‘on‐ramp’ programmes versus advice‐first; integrated medication–exercise co‐management versus parallel provision; and commissioning approaches that fund equity supports versus those that do not. We need to know what the minimum viable service looks like in under‐resourced settings, how to train the workforce at scale, and how to build community partnerships that patients will actually use.

If we want to improve life expectancy in general and at birth, we should stop asking patients with SMI to be the implementation strategy. The responsibility lies with health systems: make physical activity the default, design it for real‐world constraints, staff it properly, co‐manage it with pharmacotherapy and audit it like any other core treatment. That is the ‘how’ readers are looking for, and it is where the next decade of progress will be made.

## AUTHOR CONTRIBUTIONS

Mikel L. Sáez de Asteasu was involved in the conceptualisation, methodology and first draft writing. Mikel Izquierdo was involved in the conceptualisation, methodology and editing process. Simon Rosenbaum was involved in the conceptualisation and editing process.

## FUNDING

This work received no specific funding.

## ETHICS STATEMENT

This forum article does not report original human or animal research and therefore does not require ethical approval.

## Data Availability

Data sharing is not applicable to this article as no new data were created or analysed in this study.
